# Polyradiculoneuritis revealing an acute monoblastic leukemia 5

**Published:** 2010-06-01

**Authors:** Yahya Hsaini, Wafa Allam, Hassan Errihani

**Affiliations:** 1 Departement of Neurology, Military Hospital Med V Rabat, Morocco; 2 Departement of Medical Oncology, National Institute of Oncology Rabat, Morocco

**Keywords:** Morroco, acute monoblastic leukemia, polyradiculoneuritis, Guillain-Barre

## Abstract

Acute polyradiculoneuritis has been frequently reported in association with malignant disorders, especially those of the lymphoid system. To date, there have been no reported cases of acute monoblastic leukemia associated with this polyradiculopathy. The authors tell us about a very rare case of leukemia presenting as acute monoblastic leukemia 5 (AML5) in a 28 years old patient from Morocco.

## Letter to the editor

Acute polyradiculoneuritis has been frequently reported in association with malignant disorders, especially those of the lymphoid system. [1]. To date, there have been no reported cases of acute monoblastic leukemia associated with this polyradiculopathy.

A 28 year old male, with a history of angina treated one month before his admission, was hospitalized for functional symmetrical lower  limb impotency that begun 15 days before without urinary, respiratory, swallowing or spinal symptoms. Physical exam revealed an alert, afebrile, and well oriented patient with flexible neck. Cranial nerves were normal. He had a flaccid quadriparesis and absent tendon reflexes. There was no disorder in the appreciation of vibration and proprioception.

Clinical examination also found purpurea, bilateral superficial inguinal nodes, and lingual papillae hypertrophy. The electromyogram was in favour of a motor axonal polyradiculoneuropathy. In fact, nerve potential studies revealed a normal motor and sensory conduction velocity of all peripheral nerves, with prolonged distal latencies and marked reduction of proximal and distal motor potential amplitude of fibulae and tibialis nerve. F responses were unobtainable in lower limbs.

WBC count in blood was at 43.4 109/l. Spinal fluid examination showed 76 monoblasts with normal chemistry. LDH levels were 2698UI / l. Viral serology (HIV, VDRL, TPHA, HBV, HCV) was negative.

Cytological analysis of blast cells and cytochemical study were performed and made a definite diagnosis of an acute monoblastic leukaemia 5a (AML5a). The patient unfortunately died the next day in a context of a disseminated intravascular coagulation.

Our patient fulfils the well-established diagnostic criteria for acute polyradiculoneuritis; he developed proximal and distal quadriparesis and areflexia in 10 days. Electroneuromyogram findings confirmed symmetrical character of the neuropathy. To our best knowledge, prior cases have also been reported where leukemia has presented with acute polyradiculoreuritis [2,3], but never AML5, therefore our case is the first one illustrating this association. The mechanism of this attack may be by tumour infiltration, autoimmune disorder, neurotoxicity due to chemotherapy, or vasculitis [4]. The existence of blasts in the spinal fluid in our patient favors the tumour infiltration theory. The prognosis of this association seems more related to the hemopathy than to the neurological status [5]. Advanced age, AML 5, a high rate LDH are poor prognosis factors [6].

## Competing interests

The authors report no conflicts of interests. The authors alone are responsible for the content and writing of the paper. Authors have equally contributed to this paper.
